# Self-confidence and anxiety in clinical decision-making among nursing students during high-fidelity simulation training: a longitudinal study

**DOI:** 10.1186/s41077-026-00445-8

**Published:** 2026-05-21

**Authors:** Geert Van de Weyer, Filip Haegdorens, Senne Vleminckx, Deborah Hilderson, Erik Franck

**Affiliations:** 1 Karel de Grote University of Applied Sciences and Arts, Antwerp, Belgium; 2 Centre for Research and Innovation in Care (CRIC), Workforce management, Health Systems and Outcome Research in Care Group, Antwerp, Belgium

**Keywords:** Anxiety, Clinical decision-making, High-fidelity simulation, Nursing student, Self-confidence

## Abstract

**Background:**

Clinical decision-making is a core nursing competency that develops progressively through education and practice. However, nursing students frequently report reduced self-confidence and increased anxiety when required to make clinical decisions. High-fidelity simulation (HFS) provides a safe, realistic environment to practice clinical decision-making and may influence these psychological determinants. Longitudinal studies on how repeated exposure to HFS affects anxiety and self-confidence across an entire nursing curriculum remain limited.

**Methods:**

A four-year longitudinal study examined changes in nursing students’ self-confidence and anxiety in clinical decision-making during repeated annual exposure to HFS within a single cohort. A total of 143 students completed pre- and post-intervention questionnaires using the *Nursing Anxiety and Self-Confidence with Clinical Decision-Making—High-Fidelity Simulation scale (NASC-CDM-HFS)*. Linear mixed-effects models with random intercepts for participants examined year-by-year changes in self-confidence and anxiety, adjusted for age and prior healthcare experience. The anxiety models were further adjusted for baseline self-confidence levels.

**Results:**

Pre-intervention self-confidence increased naturally across the program, whereas pre-intervention anxiety declined, indicating growing familiarity with clinical practice. After participating in HFS, significant improvements were observed during the first year. Self-confidence increased (*b* = 6.64, 95% CI [4.17, 9.11]) and anxiety decreased (*b* = –2.90, 95% CI [–5.27, –0.68]). In subsequent years (Y2–Y4), HFS produced no additional statistically significant effects.

**Conclusion:**

High-fidelity simulation enhancess self-confidence and reduces anxiety during clinical decision-making among novice nursing students. However, significant effects were only found during the first training year. This suggests that the psychological impact of HFS is strongest when students are exposed to simulationtraining for a first time. To repeat these psychological benefits throughout the curriculum, simulation activities should be progressively adapted in complexity, integrated more frequently, and aligned with students’ advancing clinical competencies.

**Supplementary Information:**

The online version contains supplementary material available at 10.1186/s41077-026-00445-8.

## Introduction

Clinical decision-making, a foundational element in nursing practice, involves evaluating patient conditions, considering potential interventions and selecting appropriate actions [1, 2]. Despite its importance, nursing students often experience reduced self-confidence and increased anxiety, key psychological factors that can influence clinical decision-making [3, 4]. Self-confidence, defined as a person’s belief in their ability to perform tasks or achieve goals, functions as a key enabling factor, allowing students to trust their judgment, apply theoretical knowledge effectively, and remain competent under pressure [4]. In contrast, anxiety is an emotional state characterized by tension, apprehensive thoughts, and physiological responses such as an increased heart rate, which can impair critical thinking and problem-solving abilities [5]. These effects hinder the recall of important information essential for effective clinical decision-making [3].

Simulation-based training has been introduced in nursing education to bridge the gap between theoretical knowledge and clinical practice. It encompasses various modalities, such as low-, medium-, and high-fidelity mannequin-based simulations, virtual reality, or standardized patient encounters, each serving specific pedagogical goals. Among these modalities, High-Fidelity Simulation (HFS) represents the most immersive form, providing an advanced, realistic environment supported by life-size mannequins, dynamic physiological responses, and interactive clinical scenarios [6].

Whereas modality describes the simulation type, fidelity refers to the degree of realism achieved within a simulation, encompassing physical, cognitive, psychomotor, and psychological dimensions. Physical fidelity captures how accurately the equipment and setting reproduce real clinical contexts; cognitive fidelity reflects the extent to which clinical reasoning, decision-making, and cue recognition are replicated; psychomotor fidelity concerns the realism of manual and procedural skills; and psychological fidelity addresses the authenticity of emotional and interpersonal experiences, including teamwork and stress [7].

By combining these dimensions, HFS enables nursing students to practice complex clinical decision-making in a safe environment, fostering the integration of technical and non-technical competencies. Importantly, HFS triggers emotions comparable to those experienced in real clinical situations, making it particularly effective for exploring psychological factors such as self-confidence and anxiety in relation to performance and learning outcomes [8, 9]. Previous research broadly established the educational value of HFS in enhancing technical and non-technical skills [10–12]. However, fewer studies have explicitly examined its psychological impact in the context of clinical decision-making. Self-confidence and anxiety are not only general learning factors but directly shape the quality of decision-making under pressure, influencing how nursing students gather information, weigh options, and implement clinical decision-making [13].

Self-confidence and anxiety play crucial roles in shaping how nursing students engage with and benefit from HFS. Despite the recognized benefits of HFS, most research examined cross-sectional outcomes, with limited exploration of its sustained effects on anxiety and self-confidence throughout a nursing program. A longitudinal approach provides a better understanding of how HFS influences these psychological factors in the long term. This study addresses this gap by examining the longitudinal impact of high-fidelity simulation training on nursing students’ self-confidence and anxiety in clinical decision-making throughout their undergraduate education. By evaluating these dynamics, the study seeks to provide deeper insights into the role of high-fidelity simulation in enhancing nursing education and preparing students for clinical practice.

## Methods

### Study design

This single-center longitudinal study employed repeated pre- and post-test measurements to assess self-confidence and anxiety in clinical decision-making during high-fidelity simulation (HFS) in the same cohort of Bachelor nursing students.

The cohort of nursing students received one structured HFS session per academic year. The longitudinal design therefore reflects repeated annual exposure to HFS within the same cohort rather than a cumulative or intensified intervention model. As such, the study examines year-specific intervention effects within a developmental trajectory.

### Participants

The study was conducted for 4 consecutive years in the Bachelor of Nursing program at Karel de Grote University of Applied Sciences and Arts in Antwerp, Belgium. In the first year, a convenience sample of first-year nursing students was selected for participation. All eligible students were informed about the study's objectives. Only those who provided written informed consent were included in the four-year longitudinal study. An a priori power analysis was conducted using G*Power 3.1 to estimate the minimum required sample size for detecting a significant within-subject effect (d = 0.80, α = 0.05) in paired pre–post comparisons [14, 15]. This approach indicated that 52 participants would provide adequate statistical power. Although the final analysis employed linear mixed-effects models (LMM) to account for repeated measures and missing data, this initial calculation served as a conservative estimate of the required sample size.

### High-fidelity simulation

High-fidelity simulation training was implemented as an intervention within the four-year curriculum of the Bachelor of Nursing program, requiring each student to participate in one HFS session per academic year. These sessions were organized exclusively with students from the same year group. Each session consisted of approximately ten students, lasted for four hours, and comprised three scenarios. The focus and complexity of the scenarios increased progressively across the program: in the first year, scenarios primarily addressed communication-focused situations; in the second and third year, they centered on deteriorating patient scenarios requiring increasing levels of clinical reasoning and teamwork; in the fourth year, the emphasis shifted to acute care situations involving advanced decision-making under pressure. A detailed overview of the simulation sequence and its alignment with clinical placements is provided in Appendix 1.

The simulations were conducted exclusively using high-fidelity mannequins, which are lifelike, computer-controlled models capable of realistically mimicking physiological responses such as blood pressure, lung sounds, thoracic expansion and pupillary reactions.

Throughout the four-year program, only high-fidelity mannequins were used; standardized patients were not included. In each scenario, three students participated: two assumed the role of nursing students, while the third acted as a family member of the mannequin. The remaining students observed the scenario via live stream from another room.

After each 15-min scenario, all students, both participants and observers, took part in a structured group debriefing led by an EuSim simulator instructor who was a course-certified faculty member. The debriefing lasted 45 min, after which three other students engaged in the next scenario. This process continued, with each scenario followed by a full-group debriefing until all students had assumed the role of nursing student in a scenario.

The scenarios increased in cognitive, psychomotor, and metacognitive complexity throughout the four-year curriculum. In the first year, scenarios were intentionally less complex, allowing students to develop foundational cognitive and psychomotor competencies in a simulated hospital environment. These cases, such as a patient refusing the insertion of a nasogastric tube or displaying abnormal vital signs requiring immediate notification of a physician by phone, emphasized core communication and clinical reasoning, providing a safe introduction to clinical decision-making.

By the second year, scenarios incorporated greater cognitive and metacognitive complexity, primarily through interactions with patients’ families. For example, a family member might object to handing over the patient’s home medication to the nurse, fearing its loss based on a prior hospitalization experience. Such situations required students to integrate emotional intelligence, situational awareness, and professional communication while safeguarding patient care.

In the third year, the focus shifted toward managing unexpected and dynamic situations, introducing higher cognitive and psychomotor complexity. One scenario featured a hypoglycemic patient who fell out of bed, requiring rapid assessment and interventions to manage the situation and mitigate complications or injuries. These scenarios tested their ability to integrate theoretical knowledge with practical action in unpredictable conditions, fostering clinical reasoning, prioritization, and decision-making.

In the final year, the focus of HFS extended beyond non-technical skills to the integration of theoretical and clinical knowledge, leadership, teamwork, and situational awareness gained throughout the curriculum. Some scenarios were conducted in non-traditional simulation environments, such as the auditorium. For instance, final-year students, acting as nursing members of the emergency response team, provided immediate care to the individual who had collapsed on a staircase or between a row of seats, using a monitor-defibrillator to assess the situation and deliver shocks or other nursing-related interventions as required. These advanced scenarios demanded clinical expertise, coordination, and emotional regulation, requiring students to draw on their accumulated theoretical knowledge and clinical experience.

### Instrument

The Dutch Nursing Anxiety and Self-Confidence with Clinical Decision-Making for High-Fidelity Simulation (NASC-CDM-HFS) is a validated self-report questionnaire based on White’s NASC-CDM [16]. This questionnaire is specifically designed to measure nursing students’ self-confidence and anxiety during clinical decision-making in simulation training. It consists of two subscales: self-confidence and anxiety. Each subscale contains 19 items rated on a 6-point Likert scale, ranging from 1 (“not at all”) to 6 (“completely”) in agreement with a given statement [17].

A previous study, evaluated the reliability and validity of the Dutch NASC-CDM-HFS, demonstrating its factor structure and high internal consistency for both subscales: self-confidence (α = 0.955) and anxiety (α = 0.956) [17]. Explanatory Factor Analysis for the self-confidence subscale identified three factors: Factor 1, using resources to gather information and listening fully, Factor 2, using information to see the big picture (*n* = 6), and Factor 3, knowing and acting (*n* = 6). For the anxiety subscale, two factors were identified: Factor 1, gathering information, applying knowledge for decision-making, and initiating action (*n* = 12), and Factor 2, using resources to gather information and listening fully (*n* = 7).

### Data collection

**Fig. 1:**
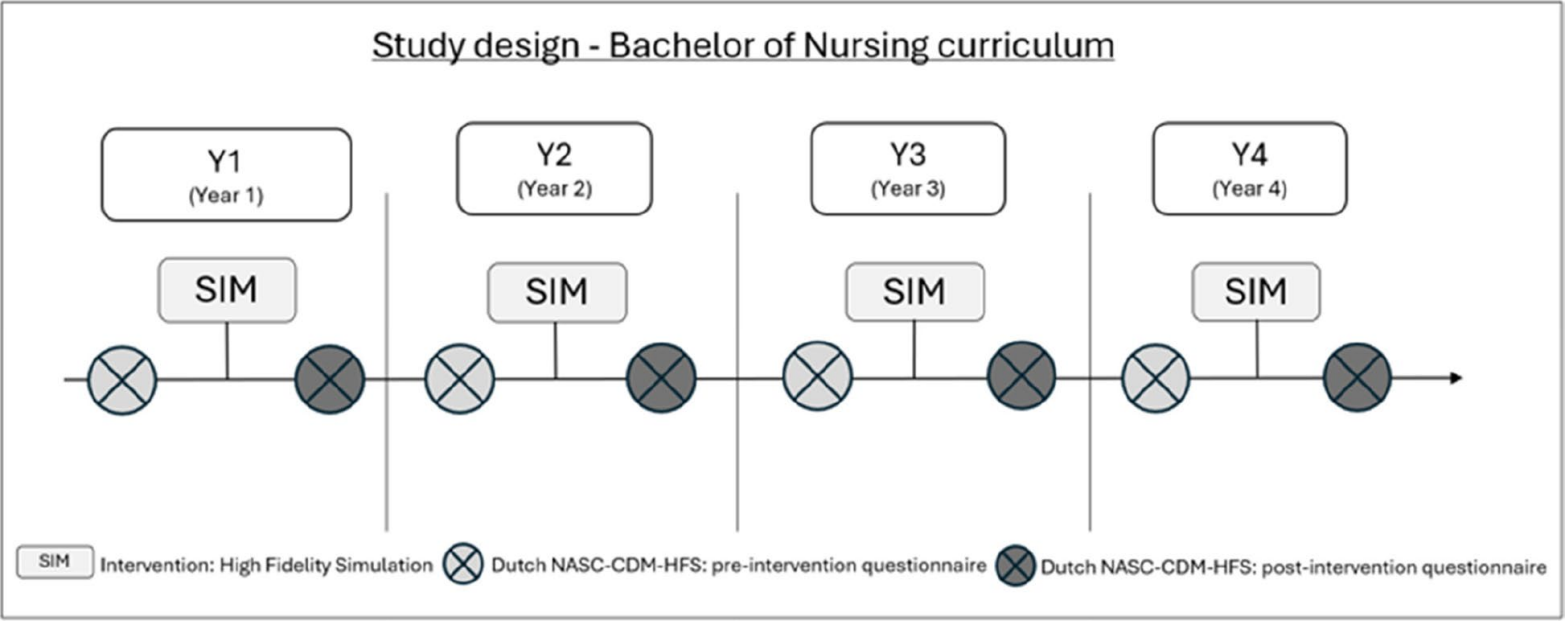
Study design Bachelor of Nursing curriculum

Data collection spanned the four-year Bachelor of Nursing curriculum. The same longitudinal cohort of nursing students was surveyed using a pre- and post-intervention questionnaire in every year (Fig. 1). In the first year (Y1), data were collected in May 2018. Second year (Y2) data were collected between November and December 2018. In the third year (Y3), data collection took place from September to November 2019. Finally, the fourth year (Y4) data collection occurred in May 2021 (Fig. 1).

All the data were collected using the electronic survey platform Qualtrics. The HFS center coordinator, who served as the principal investigator, was present during the survey sessions to address participant inquiries.

Participants completed the pre-intervention questionnaire one week before the start of the HFS. This included demographic information, their self-reported self-confidence and anxiety during clinical decision-making in HFS. Immediately following the HFS, students completed the post-intervention questionnaire. No personal data was collected. A unique student number was used to compare pre- and post-intervention results after pseudonymization.

### Data analysis

The analytical sample included students who participated in Year 1 and were present in at least one subsequent year (Years 2–4). Sum scores were calculated for both the self-confidence and anxiety subscales by summing all 19 items within each scale. Students with missing scores were excluded on a pairwise basis, maximizing the use of available data.

Linear mixed-effects models were employed to account for the hierarchical structure of the data, where repeated measurements (Level 1) were nested within students (Level 2). Separate models were fitted for self-confidence and anxiety as dependent variables. The models included year (categorical: Years 1–4, with Year 1 as reference), time point (pre or post), and their interaction as fixed effects. The interaction term tested whether the effect of HFS training differed across academic years. To control for potential confounding variables, the self-confidence model included age (categorical: 18–20, 21–25, 26 + years) and prior healthcare work experience (binary: yes/no) as covariates. The anxiety model included these same covariates plus baseline self-confidence scores. A random intercept was specified for each student to account for baseline individual differences and the repeated nature of measurements within the same student over time.

The **"**Net Intervention Effect**"** was derived from these adjusted models as the estimated change from pre to post measurement (main effect of time point), controlling for the natural progression across academic years (year effects) and relevant covariates. For Years 2–4, the net intervention effect was calculated by combining the main time effect with the respective year-by-time interaction term. This net effect represents the intervention-specific change in the outcome variable beyond natural year-related progression and individual characteristics.

Model fit was evaluated using log-likelihood and Akaike Information Criterion (AIC) [18]. Model assumptions were assessed through diagnostic plots. Model assumptions were formally tested using multiple diagnostic procedures. Normality of residuals was assessed using the Shapiro–Wilk test, and skewness, and kurtosis statistics [19, 20]. Homoscedasticity was evaluated using the modified Breusch-Pagan test and Levene's test across academic years [21, 22]. In cases where assumption violations were detected, bootstrapped confidence intervals were computed to ensure robust inference [23]. To assess the potential impact of missing data and attrition, a sensitivity analysis was conducted by re-fitting the models using only the 40 students who provided complete data across all four years and both measurement occasions. Results from the complete-case analysis were compared with those from the full available-data analysis to evaluate the robustness of findings.

Statistical significance was determined using z-tests for fixed effects, with α = 0.05. The main effect of time point represented the HFS training effect in Year 1 (change from pre to post). Year-specific training effects in Years 2–4 were calculated by combining the main time effect with the respective interaction terms. For self-confidence, positive coefficients indicate improvement from pre to post, while for anxiety, negative coefficients indicate reduction (improvement). All statistical analyses were conducted using Python 3.11.11 with the following packages: pandas 2.2.2 (data manipulation) [24], numpy 1.26.4 (numerical operations) [25], statsmodels 0.14.4 (mixed-effects models) [26], matplotlib 3.10.0 (visualization) [27], and scipy 1.13.1 (statistical functions) [28]. Models were estimated using restricted maximum likelihood (REML) [29].

## Results

### General

The study followed a cohort of nursing students throughout their four-year curriculum, evaluating their anxiety and self-confidence in clinical decision-making each year before and after HFS. Data were collected at four measurement points: Y1, Y2, Y3, and Y4 (Fig. 1).

**Fig. 2:**
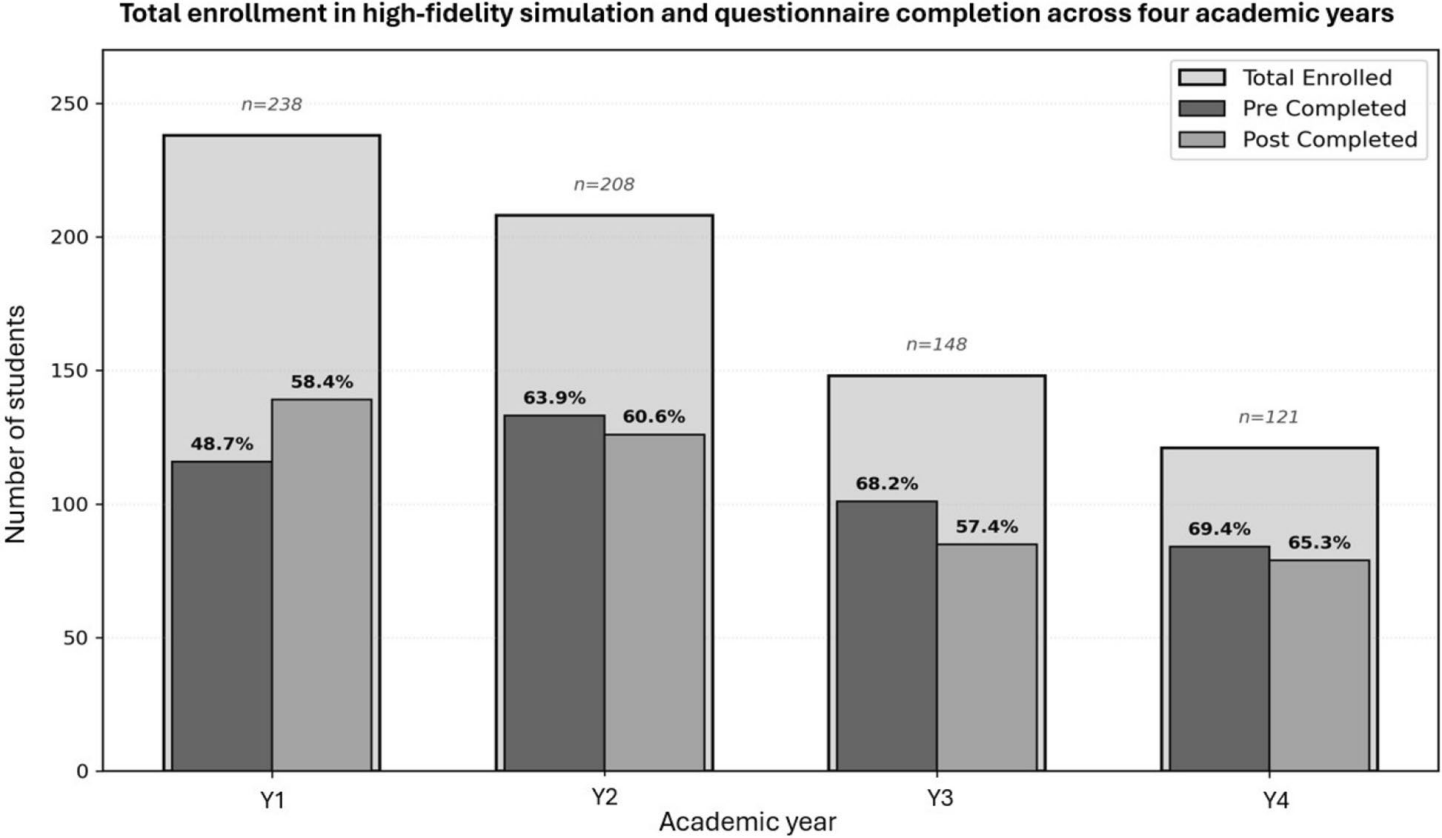
Total enrollment of nursing students in high fidelity simulation training and the questionnaire completion across the Bachelor of Nursing curriculum

Student participation in HFS showed a gradual decrease across the four academic years, from 238 students in year 1 (Y1) to 121 in year 4 (Y4). However, engagement in completing the pre- and post-intervention questionnaires remained stable. In Year 1, 48.7% of participants completed the pre-intervention and 58.4% the post-intervention questionnaire. Completion rates were 63.9% and 60.6% in Year 2, 68.2% and 57.4% in Year 3, and 69.4% and 65.3% in Year 4, respectively. These findings indicate that, despite a lower number of participants in HFS over time, response rates among participants remained consistent throughout the study period (Fig. 2).

### Demographics

At baseline (Y1), 143 nursing students participated in the study. The mean age was 20.10 years (SD = 4.8; median = 19, IQR = 18.0–20.0). Most participants were female (89.6%), and nearly one quarter (24.3%) reported prior work experience in the healthcare sector. Additional demographic characteristics are presented in Table 1.

**Table 1:**
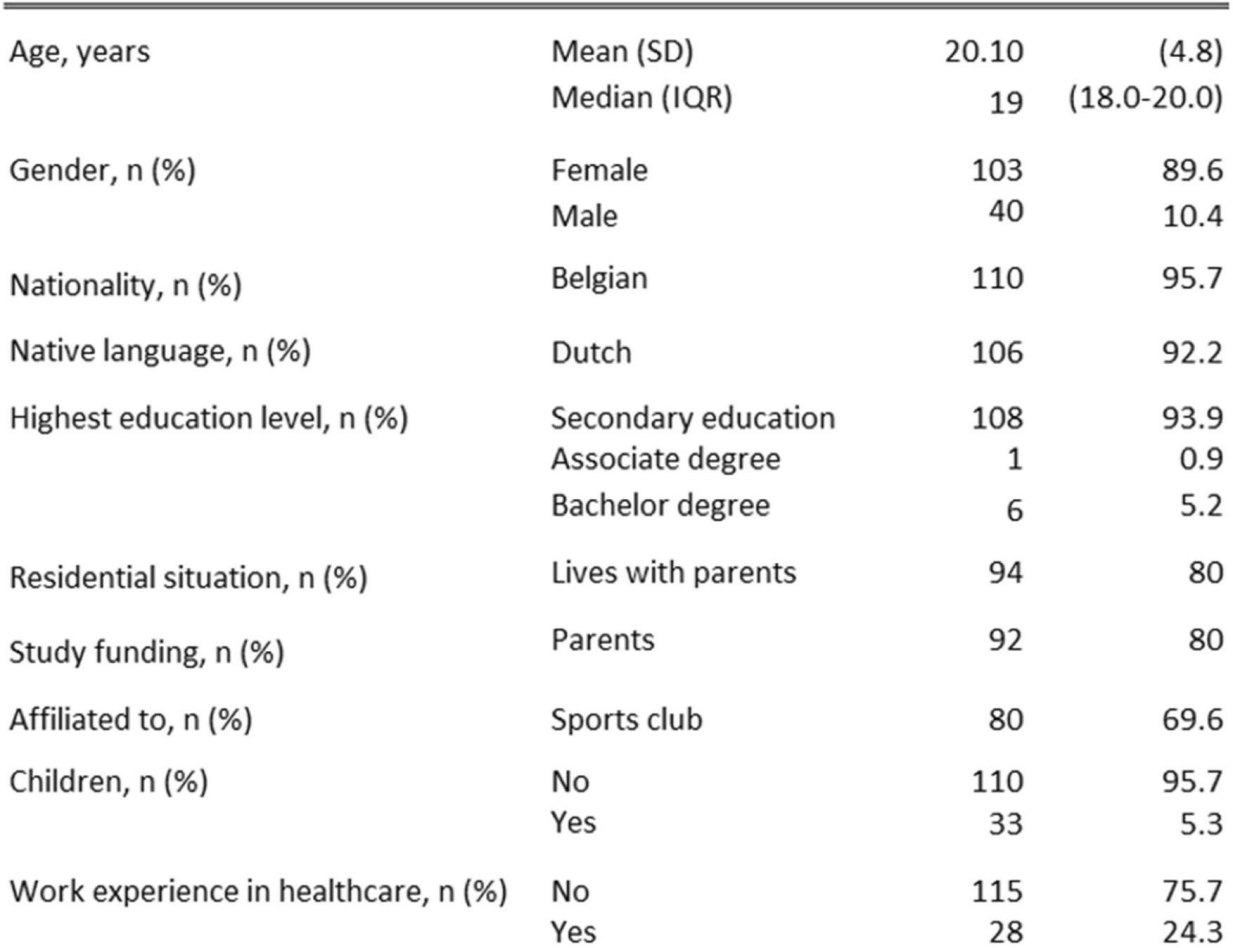
Demographic characteristics (*N* = 143)

Relevant demographic characteristic of the study participants

*SD* Standard Deviation with 95% CI, *IQR* Interquartile range

### Self-confidence

Linear mixed-effects models examined intervention effects on self-confidence across four academic years (*n* = 853 observations from 143 students, mean observations per student = 6.0). The baseline self-confidence score (intercept) was 66.47 (SE = 1.36, 95% CI [63.80, 69.13], *p* < 0.001). Model assumptions of normality and homoscedasticity were met, validating inferential testing. Detailed model estimates are presented in Table 1.

Significant year effects were observed, indicating a progressive natural self-confidence gain across the curriculum (Y2: b = 3.96, *p* = 0.002; Y3: b = 4.42, *p* = 0.003; Y4: b = 10.77, *p* < 0.001). This natural increase in self-confidence reflects the gradual increase in pre-intervention self-confidence scores over time.

The pre–post intervention effect varied significantly across years. Immediately following HFS, a significant improvement in self-confidence was found in Year 1 (b = 6.64, 95% CI [4.17, 9.12], *p* < 0.001, β = 0.223). Examining the adjusted net intervention effects across years, no additional significant pre–post improvements were observed beyond Year 1: Year 2 (b = 0.63, 95% CI [−3.65, 4.91]), Year 3 (b = −0.41, 95% CI [−4.93, 4.11]), and Year 4 (b = 2.10, 95% CI [−2.54, 6.74]). Students with prior healthcare work experience reported significantly lower post-intervention self-confidence (b = –2.48, 95% CI [−4.75, –0.21], p = 0.032, β = –0.223). Individual developmental trajectories across years are visualized in Appendix 2a, Appendix 2b presents the mean self-confidence scores pre- and post-HFS across the curriculum, along with the adjusted net intervention effects across all academic years (Table 2).

**Table 2:**
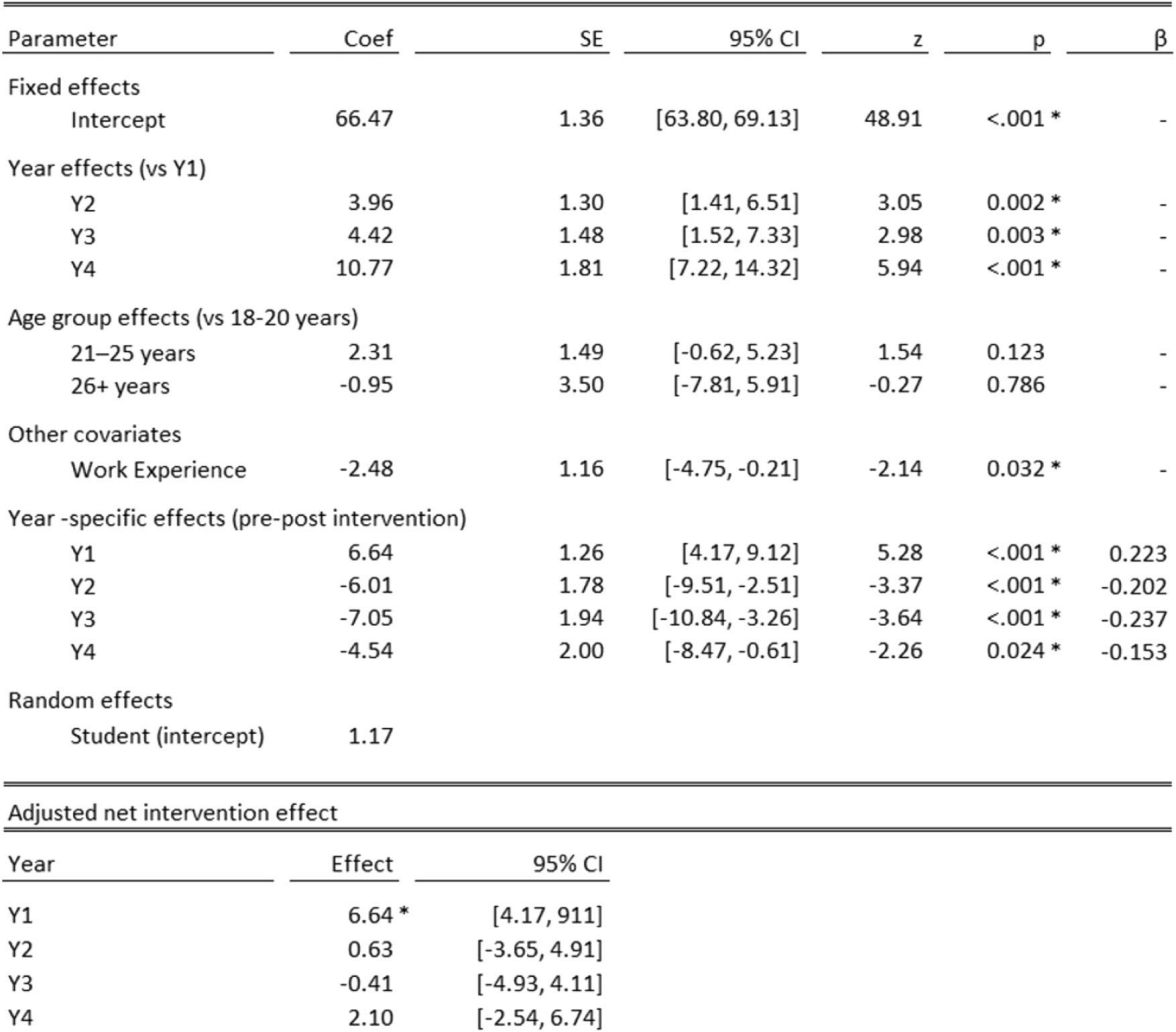
Linear mixed-effects models-intervention effects on self-confidence scores (*N* = 143)

Statistical test-Linear mixed-effects models, accounts for repeated measure within students

β standardized coefficients (- = standardized coefficient not meaningful for categorical predictors); Y = academic years Bachelor of Nursing; * = significant effect (95% CI excludes 0)

### Anxiety

For anxiety, LMM examined changes across the four academic years while accounting for repeated measurements within students (*n* = 845 observations from 143 students; mean observations per student = 5.9). The baseline anxiety score (intercept) was 83.23 (SE = 2.43, 95% CI [78.47, 87.99], *p* < 0.001). Detailed model estimates are presented in Table 3.

**Table 3:**
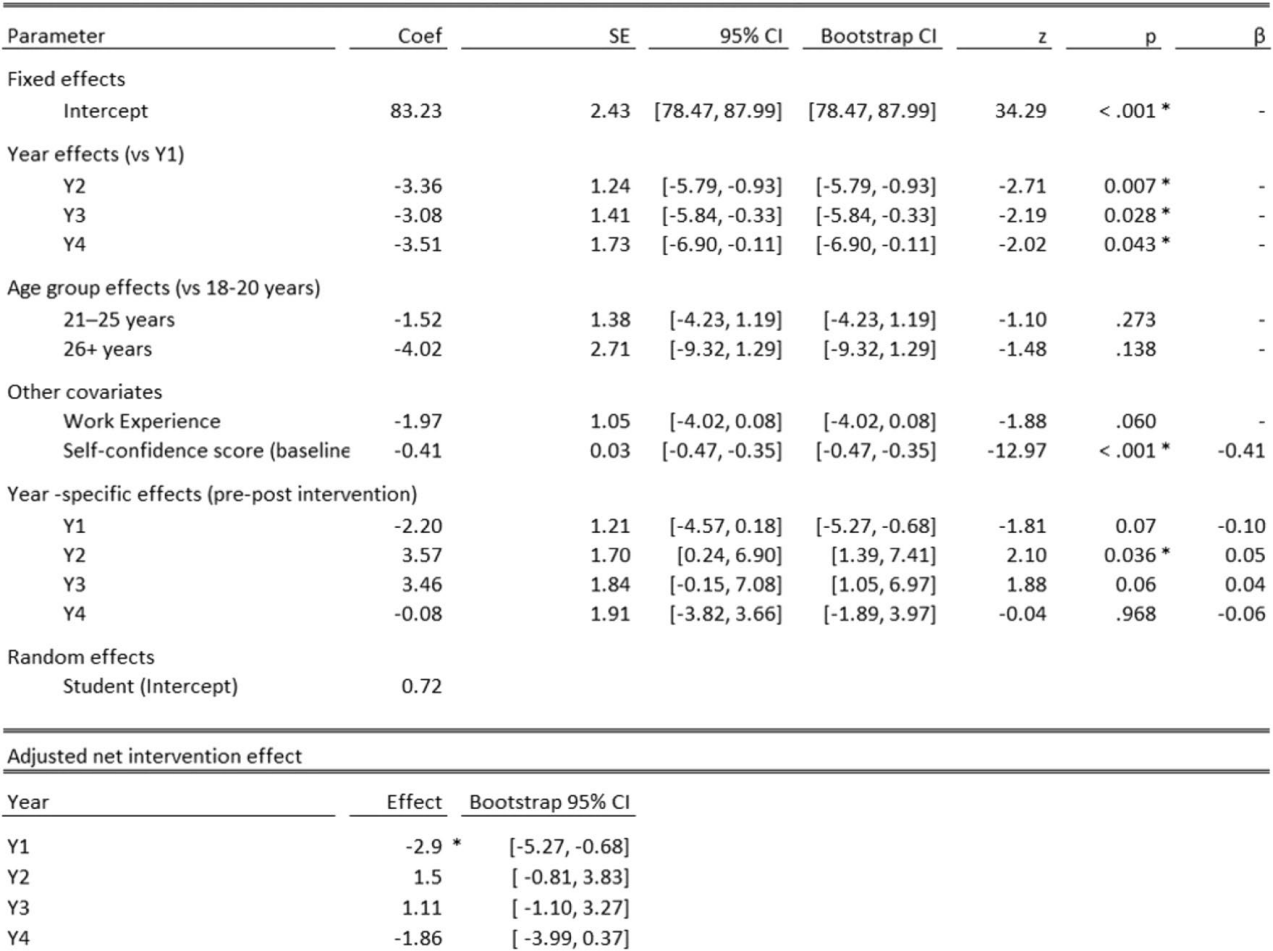
Linear mixed-effects models- intervention effects on anxiety scores (*N* = 143)

Statistical test- Linear mixed-effects models; Bootstrap Cis: 1, 000 iterations; cluster-aware bootstrap maintains within-student correlation; β = standardized coefficients (- = standardized coefficient not meaningful for categorical predictors); Y = academic years of Bachelor of Nursing; * = significant effect (bootstrap CI excludes 0)

Significant year effects were identified after controlling for age, prior healthcare work experience, and baseline self-confidence, indicating a progressive natural anxiety decrease throughout the curriculum compared with Year 1 (Y2: b = –3.36, *p* = 0.007; Y3: b = –3.08, *p* = 0.028; Y4: b = –3.51, *p* = 0.043). This natural anxiety change reflects the gradual reduction in pre-intervention anxiety over time, representing developmental trends independent of the intervention.

Model diagnostics revealed violations of normality (Shapiro–Wilk W = 0.97, *p* < 0.001) and homoscedasticity assumptions (Breusch-Pagan χ^2^ = 51.64, *p* < 0.001); therefore, bootstrap confidence intervals (1,000 iterations) were used for inference on intervention effects. The pre–post intervention effect of HFS varied across academic years. Immediately following HFS, a significant decrease in anxiety was observed in Year 1 (b = –2.90, bootstrap 95% CI [–5.27, –0.68], β = −0.10). However, when controlling for natural developmental trends and baseline covariates, no significant intervention effects were observed in subsequent years: Year 2 (b = 1.50, bootstrap 95% CI [−0.81, 3.83]), Year 3 (b = 1.11, bootstrap 95% CI [−1.10, 3.27]), and Year 4 (b = −1.86, bootstrap 95% CI [−3.99, 0.37]). This pattern suggests the intervention produced an immediate anxiety-reducing benefit specific to Year 1 students.

Among covariates, baseline self-confidence emerged as a strong negative predictor of anxiety (b = –0.41, 95% CI [–0.47, –0.35], *p* < 0.001, β = –0.41), indicating that higher self-confidence was consistently associated with lower anxiety scores. Appendix 3a illustrates individual developmental trajectories across years, while Appendix 3b presents mean anxiety scores before and after HFS across the curriculum, along with the adjusted net intervention effects across all academic years.

## Discussion

This longitudinal study examined changes in nursing students’ self-confidence and anxiety in clinical decision-making during repeated annual exposure to HFS within a single cohort across four academic years. The findings demonstrate that HFS positively influenced first-year nursing students’ self-confidence and anxiety, whereas these effects could not be found in later years. The results suggest that simulation exerts its strongest measurable impact during an early stage of professional development, when students are novices encountering clinical decision-making in complex situations for the first time.

Although no pre–post effects were observed in Years 2–4, this should not be interpreted as definitive evidence of absence of an intervention effect. Rather, the adjusted estimates indicate that, after accounting for year effects and covariates, no detectable pre–post changes were observed following HFS in the following years. The confidence intervals for the Year 2–4 estimates were relatively wide, suggesting statistical uncertainty.

Overall participation in HFS declined over the course of the program, however the set of students who remained included this study was relatively stable across the four years, ensuring adequate longitudinal representation. This decline was partly because not all students completed the standard four-year program trajectory; some followed individualized study paths or stopped from the nursing program altogether. Not all participants completed the questionnaires at every measurement point, resulting in partially missing data. The use of LMM was therefore appropriate, as it accounted for the hierarchical structure of repeated observations and effectively handled incomplete data without reducing statistical power or introducing bias [30]. These methodological decisions strengthen the reliability and validity of the findings.

From a psychological perspective, these findings can be interpreted through Bandura’s social cognitive framework, which centers on the concept of self-efficacy—individuals’ beliefs in their capabilities to organize and execute the actions required to manage prospective situations [31]. Bandura identifies four major processes through which self-efficacy operates: cognitive, motivational, affective, and selection mechanisms. In educational contexts, perceived efficacy influences how learners interpret challenges, regulate emotions, and sustain effort under pressure. In this study, HFS served as a mastery experience, the most powerful source of self-efficacy, by allowing students to apply theoretical knowledge in realistic yet psychologically safe environments that provide immediate feedback and visible consequences for their actions [32]. Such experiences likely strengthened students’ perceived capacity for clinical decision-making in complex situations. Although this study measured self-confidence, the construct conceptually aligns with Bandura’s task-specific self-efficacy, the belief in one’s ability to organize and execute actions required to achieve specific goals, enabling students to manage uncertainty and maintain control under pressure. These mastery experiences are known to strengthen efficacy beliefs, which in turn enhance performance and reduce anxiety [31, 33].

In later years of the curriculum, however, as students accumulate real clinical experience and become more aware of the complexity of nursing practice, these mastery effects may diminish. This attenuation of effect suggests that the psychological benefits of HFS are most pronounced when students face novel and moderately demanding situations [34, 35].

Several mechanisms may help explain the reduction in statistically detectable effects in Years 2–4. First, ceiling effects in self-confidence may have limited measurable improvement. Pre-intervention self-confidence increased progressively across academic years, suggesting that students entered later simulation sessions with already high baseline levels. As scores approach the upper limits of the scale, the potential for further measurable improvement decreases.

Second, the reduced novelty of HFS over time may have moderated changes in students’ anxiety and self-confidence during repeated exposure. In Year 1, students experience HFS as a novel, exciting and emotionally engaging learning environment, which may increase affective and motivational engagement. In later years, familiarity with HFS, expectations and debriefing may have reduced emotional arousal, leading to smaller measurable changes in anxiety and self-confidence. Similar habituation effects have been reported in experiential learning contexts where repeated exposure reduces the intensity of affective responses [36, 37].

Third, measurement-related factors may also contribute to the observed pattern. As students progress through the curriculum, their internal standards for evaluating their own performance may evolve alongside increasing theoretical knowledge, metacognitive awareness and clinical experience. This developmental shift may lead students to assess their competencies more critically over time, potentially attenuating observable changes in perceived self-confidence and anxiety despite continued skill development.

The absence of significant post-intervention improvements in self-confidence and anxiety beyond the first year may also be interpreted through Benner’s novice-to-expert framework [38, 39]. According to Benner, students evolve from novices, who rely on rule-based reasoning, to competent and proficient practitioners capable of contextual judgement and prioritization. Although HFS scenarios in this study were designed to increase in complexity across the curriculum, the incremental changes may not have been sufficient to challenge students in later years, when their developmental level advanced. When scenarios fail to match the learner’s stage of competence, cognitive engagement diminishes and simulation becomes more confirmatory than developmental [40]. This mismatch may explain why post-intervention self-confidence decreased and anxiety remained stable relative to pre-intervention levels in later years. Further progressive adaptation of scenario design is therefore essential to ensure that each stage of the curriculum exposes students to novel, uncertain, and cognitively demanding experiences that stimulate clinical reasoning and reflective capacity. Additionally, incorporating other forms of simulation, such as scenarios with standardized patients or interprofessional exercises involving medical and other healthcare students, could offer a more challenging and enriching learning experience [41].

The observed stagnation can further be understood through Kolb’s experiential learning theory [42]. In the first year, HFS likely facilitated the full experiential learning cycle: students engaged in concrete experiences, reflected on their actions, conceptualized underlying principles, and actively experimented with new strategies in subsequent scenarios. In later years, however, the learning process may have stalled after the reflective stage, with insufficient opportunities for critical conceptualization and active experimentation. Kolb’s model emphasizes that meaningful learning requires continuous movement through all four phases of the cycle. Moreover, learning is most effective when experiences are contextually rich, challenging, and accompanied by critical reflection [42]. In this respect, the debriefing process represents a key learning moment. Within the current study, debriefing sessions were standardized and facilitated by trained instructors, yet their quality and depth were not systematically assessed. Although debriefing undoubtedly fosters reflection and emotional processing [43], its pedagogical effectiveness depends on the degree to which facilitators stimulate reasoning and the re-conceptualization of experience [44, 45]. Future simulation programs should therefore aim to strengthen debriefing practices by explicitly linking them to Kolb’s reflective and conceptual phases, thereby enhancing critical thinking and supporting the consolidation of self-confidence while mitigating anxiety [46].

Taken together, these findings suggest that sustained psychological and cognitive benefits of HFS depend on a dynamic balance between challenge, reflection, and emotional safety [47]. Integrating the theoretical perspectives of Bandura, Benner, and Kolb offers a comprehensive framework for curriculum development. Future research could explore whether simulation designs that progressively increase scenario complexity aligned with learners’ developmental stages [40], incorporate opportunities for full experiential learning cycles with structured critical reflection [48] and foster environments that support mastery experiences and psychological safety may help sustain improvements in self-confidence and reduce anxiety [35]. Such approaches may help maintain the pedagogical value of simulation-based education throughout the nursing curriculum and support its impact on clinical decision-making.

While these study findings provide valuable theoretical and practical insights, certain methodological considerations should be acknowledged to contextualize the results. First, the pre-post design without a concurrent control group limits causal inference regarding intervention effects. Although we controlled for natural developmental trends and baseline covariates, we cannot definitively rule out history effects or other time-varying confounders that may have differentially affected cohorts. Second, violations of normality and homoscedasticity assumptions in the anxiety model required bootstrap confidence intervals, suggesting potential unmodeled heterogeneity in intervention response patterns. Third, the study found no consistent impact on self-confidence and anxiety beyond Year 1, which may be explained by the limited exposure to high-fidelity simulation, consisting of only one annual session with three scenarios and a debriefing. Previous research has shown that repeated simulation experiences can progressively build self-confidence among nursing students [49]. This suggests that integrating more frequent simulation sessions could better support students’ self-confidence development over time. Additionally, the study was conducted at a single educational institution, which limits the generalizability of the findings. Moreover, the exclusive use of quantitative surveys restricted insights into nursing students’ lived experiences of self-confidence and anxiety in clinical decision-making during HFS. Future research should incorporate qualitative methods, such as focus groups or semi-structured interviews, and employ randomized controlled designs to gain a deeper understanding of these experiences [50]. Integrating quantitative and qualitative approaches may further enhance the effectiveness of HFS as an authentic and psychologically supportive learning environment.

Additionally, the study relied exclusively on self-report instruments to assess psychological constructs. Although the NASC-CDM-HFS demonstrates strong psychometric properties, self-reported measures may be influenced by social desirability, impression management, or participants’ expectations regarding improvement following simulation training. Repeated measurement may also introduce response reactivity, as familiarity with questionnaire items can influence subsequent responses. Furthermore, the absence of objective behavioural or physiological indicators limited the opportunity to triangulate psychological outcomes. Future research integrating self-report measures with performance-based or behavioural assessments may provide a more comprehensive understanding of how simulation influences both perceived and actual competence development. By situating these limitations within the broader pedagogical and theoretical context, the study emphasizes the importance of methodological refinement in future research. The year-specific intervention effects of this study underscore the need to continue enhancing HFS to better address the evolving needs of nursing students in managing self-confidence and anxiety in clinical decision-making across all years of their education. Future research should prioritize randomized controlled trials across multiple institutions to enhance generalizability. Additionally, mixed-methods approaches integrating quantitative outcomes with qualitative inquiry could reveal mechanisms underlying differential intervention responses across cohorts, ultimately informing evidence-based optimization of HFS pedagogy in nursing education.

## Conclusion

This longitudinal study examined the impact of high-fidelity simulation (HFS) training on nursing students’ self-confidence and anxiety in clinical decision-making throughout their four-year undergraduate education program. The findings revealed a significant positive effect on first-year students, indicating that HFS effectively enhances self-confidence and reduces anxiety at the beginning of their nursing education. However, these effects were not repeated over time, suggesting that the impact of HFS depends on the learner’s developmental stage and the complexity of the simulation design.

Future research should explore how simulation design and curriculum integration can be optimized to sustain improvements in nursing students’ self-confidence and anxiety throughout their education, ideally using multi-institutional and experimental study designs.

## Supplementary Information

Supplementary Material 1.

## Data Availability

The datasets used and/or analyzed during the current study are available from the corresponding author on reasonable request.
