# Supplementary material for: Self-confidence and anxiety in clinical decision-making among nursing students during high-fidelity simulation training: a longitudinal study
Source: Adv Simul (Lond). 2026 May 21;11:50. doi: 10.1186/s41077-026-00445-8 (PMC13366682; doi:10.1186/s41077-026-00445-8)
Supplement: Supplementary file 1 — Supplementary Material 1. [file 41077_2026_445_MOESM1_ESM.docx]

**Appendix**

Appendix 1: Overview of simulation-based training sessions and clinical placements across the Bachelor of Nursing Curriculum at KdG – Belgium

|  | Period 1 | Period 2 | Period 3 | Period 4 |
| --- | --- | --- | --- | --- |
| Year 1 | Theory | Theory | HFS | Clinical placement |
| Year 2 | Clinical placement | HFS | Theory | Clinical placement |
| Year 3 | Clinical placement | HFS | Clinical placement | Theory |
| Year 4 | HFS | Clinical placement | Clinical placement | Clinical placement |

*Appendix 1: The Bachelor of nursing program spans four academic yeas, each divided into four curricular periods. Students alternate between theoretical coursework (Theory), High-fidelity simulation training (HFS) and clinical placements (Clinical placement). Simulation sessions are strategically positioned to enhance the transfer between theory and clinical practice. In the first year, simulation precedes the initial clinical placement, while in subsequent years, it is integrated between clinical placements.*

Appendix 2a: Effect of high-fidelity simulation on nursing students’ self-confidence
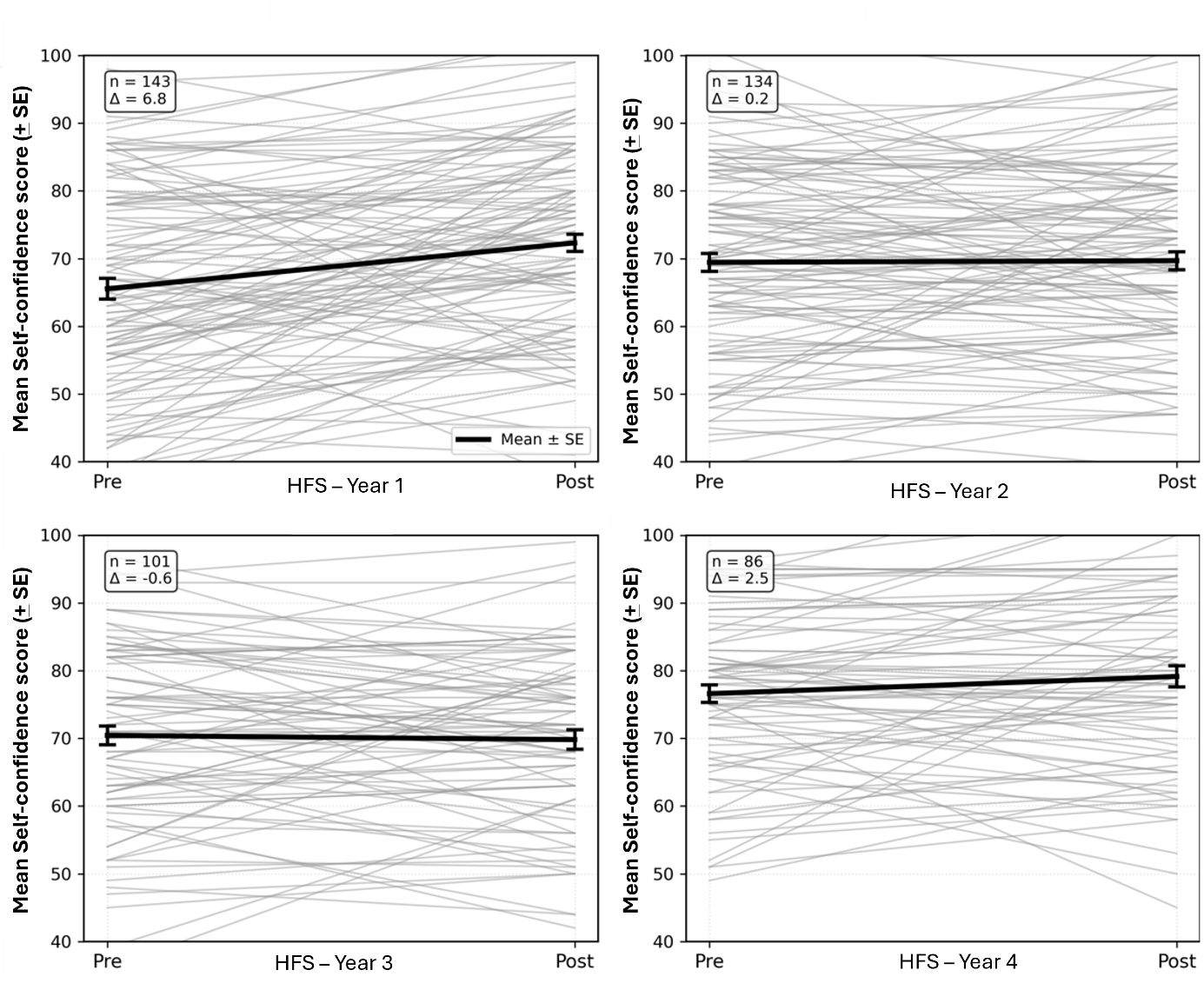
*Appendix 2a: Statistical test - Linear mixed-effects models (LMM). Mean self-confidence scores (± SE) are shown before (Pre) and after (Post) high-fidelity simulation (HFS) across four academic years. Thin grey lines represent individual participants’ trajectories, and thick black lines indicate estimated marginal means (± SE) derived from the LMM. Δ = mean pre–post differences; n = number of participants included per year.*

Appendix 2b: Effect of high-fidelity simulation on nursing students’ self-confidence


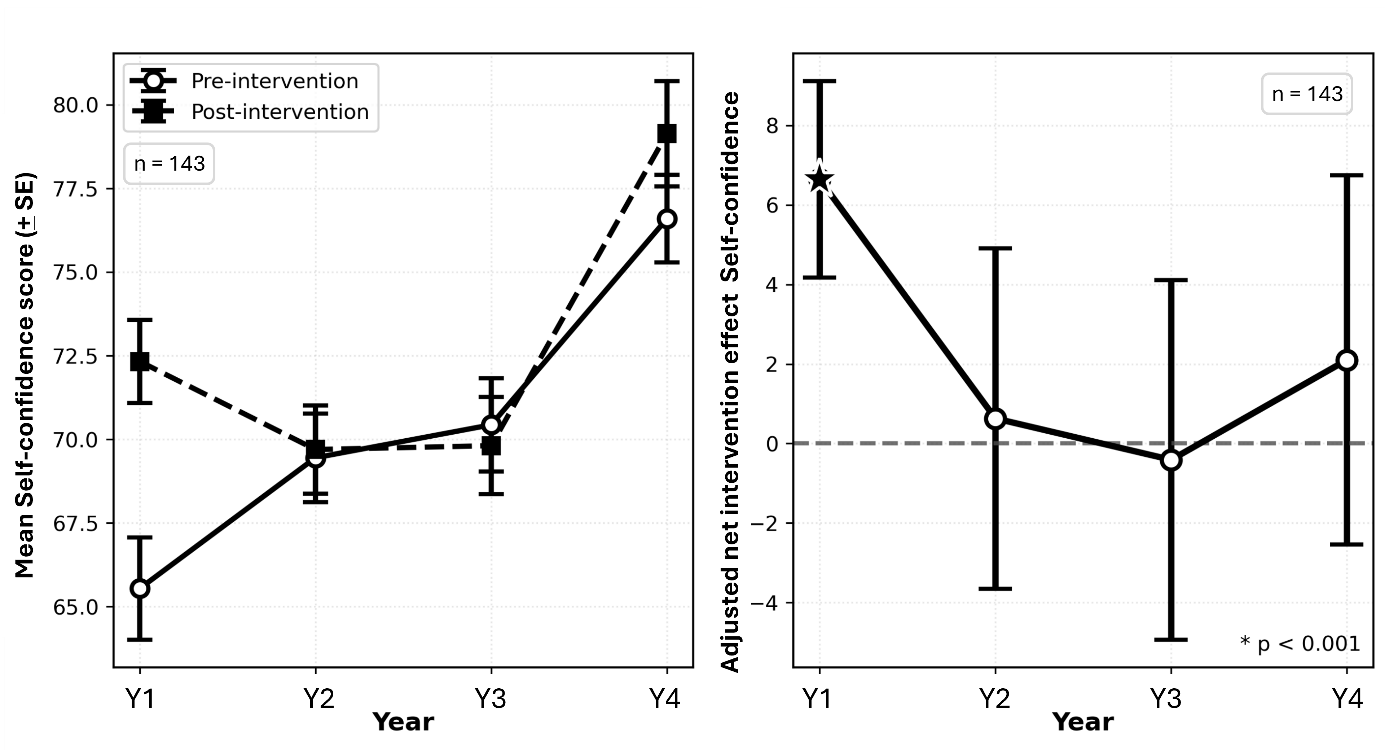
*Appendix 2b: Statistical test - Linear mixed-effects models (LMM). Left panel: Mean self-confidence scores (± SE) before (Pre) and after (Post) high-fidelity simulation (HFS) across four academic years. Right panel: Adjusted net intervention effects (± SE) on self-confidence estimated from the LMM. Δ= adjusted mean pre–post differences per year; n = total number of participants included. p < 0.001 = statistically significant effect.*

Appendix 3a: Effect of high-fidelity simulation on nursing students’ anxiety
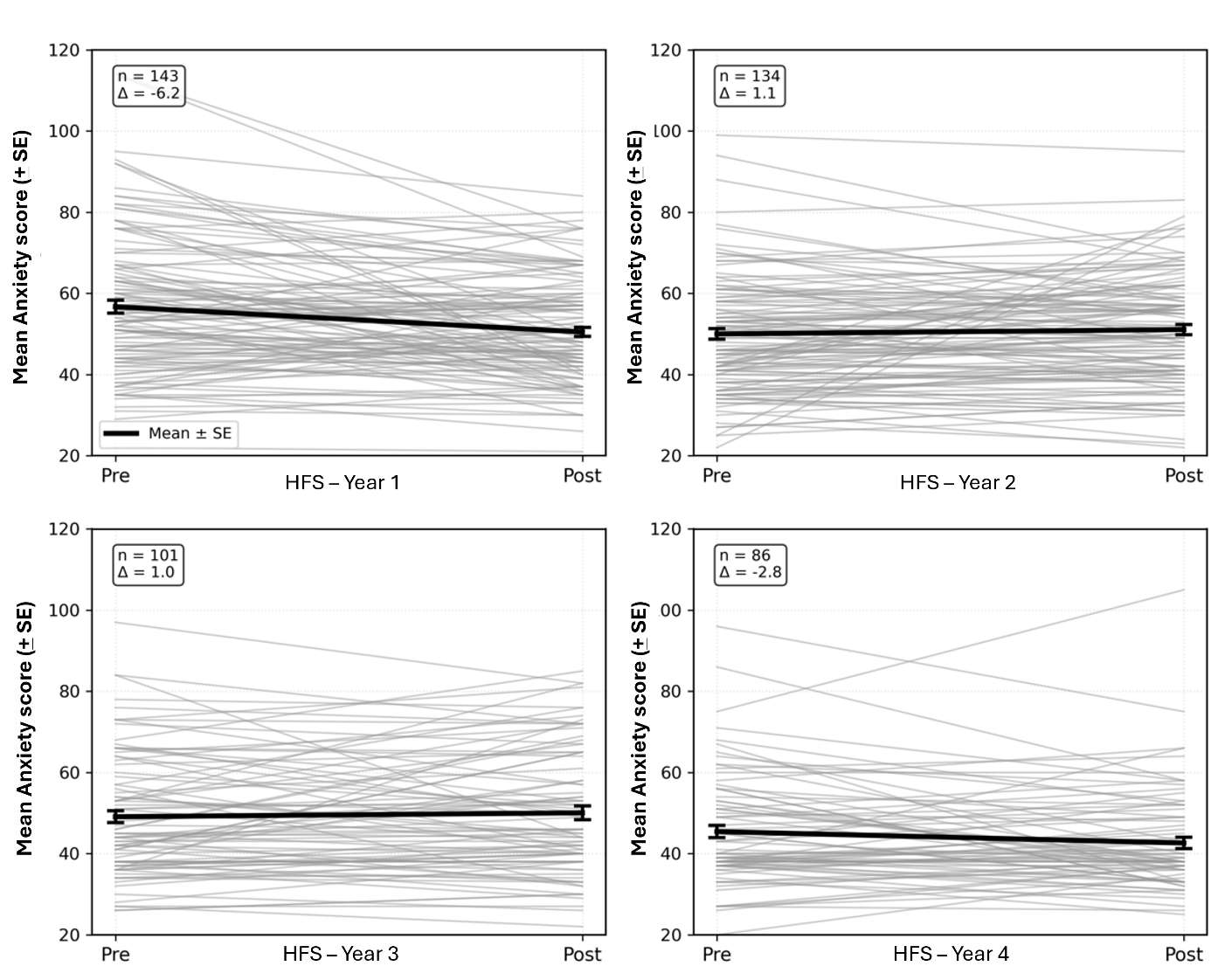

*Appendix 3a: Statistical test - Linear mixed-effects models (LMM). Mean anxiety scores (± SE) are shown before (Pre) and after (Post) high-fidelity simulation (HFS) across four academic years. Thin grey lines represent individual participants’ trajectories, and thick black lines indicate estimated marginal means (± SE) derived from the LMM. Δ = mean pre–post differences; n = number of participants included per year.*

Appendix 3b: Effect of high-fidelity simulation on nursing students’ anxiety


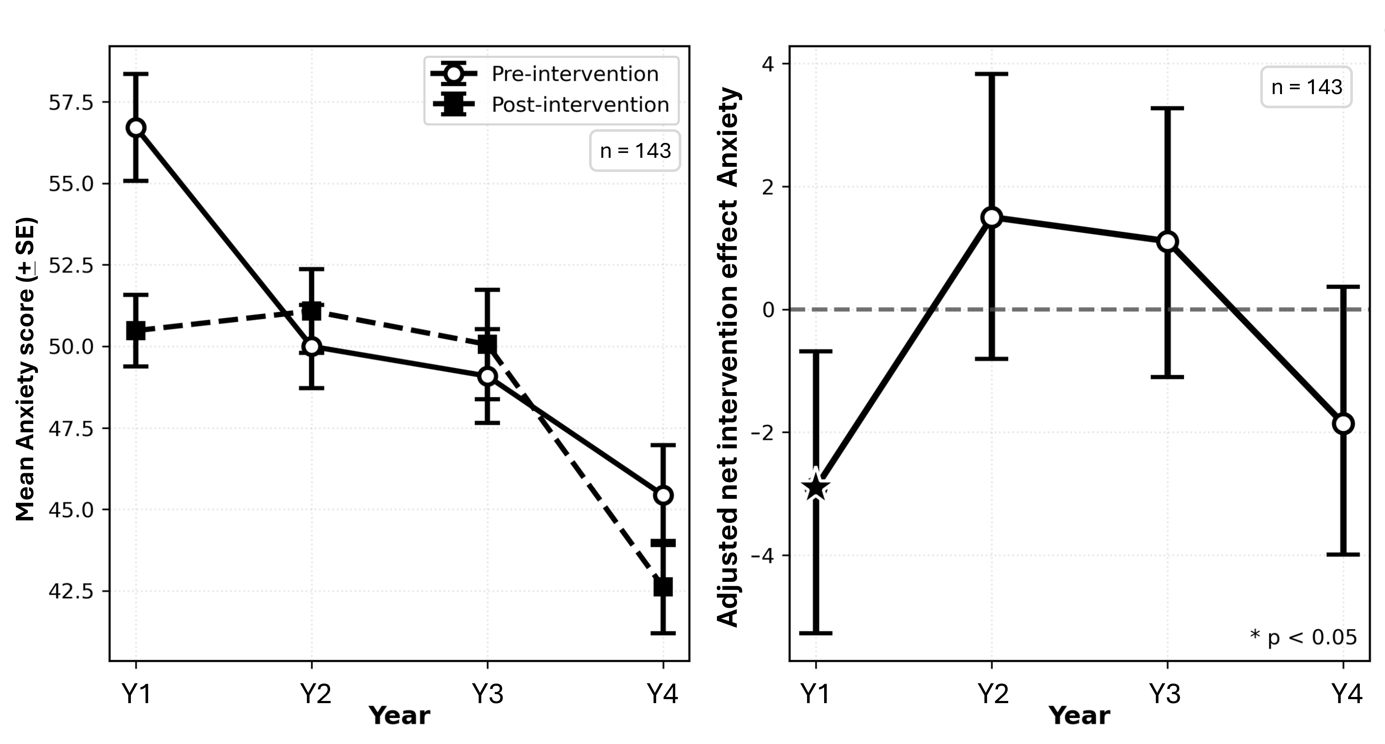

*Appendix 3b: Statistical test - Linear mixed-effects models (LMM). Left panel: Mean anxiety scores (± SE) before (Pre) and after (Post) high-fidelity simulation (HFS) across four academic years. Right panel: Adjusted net intervention effects (± SE) on self-confidence estimated from the LMM. Δ= adjusted mean pre–post differences per year; n = total number of participants included. p < 0.05 = statistically significant effect.*
